# Role of Bronchial Washing Gene Xpert in Sputum-Scarce Cases of Suspected Pulmonary Tuberculosis

**DOI:** 10.12669/pjms.35.1.107

**Published:** 2019

**Authors:** Faisal Faiyaz Zuberi, Sagheer Hussain, Sidra Hameed, Bader Faiyaz Zuberi

**Affiliations:** 1 Dr. Faisal Faiyaz Zuberi, FCPS (Med.) FCPS (Pulmonology). Ojha Institute of Chest Diseases, Dow University of Health Sciences, Karachi, Pakistan; 2 Sagheer Hussain, MBBS. Ojha Institute of Chest Diseases, Dow University of Health Sciences, Karachi, Pakistan; 3 Sidra Hameed, DTCD. Ojha Institute of Chest Diseases, Dow University of Health Sciences, Karachi, Pakistan; 4 Bader Faiyaz Zuberi, FCPS. Dow Medical College, Dow University of Health Sciences, Karachi, Pakistan

**Keywords:** Pulmonary Tuberculosis, Bronchoscopy, Sputum Smear, Bronchial Washing

## Abstract

**Objective:**

To determine the frequency of mycobacterium tuberculosis detection, in bronchial washing in sputum-scarce cases of suspected pulmonary tuberculosis.

**Methods:**

A descriptive cross-sectional study was conducted at the Ojha Institute of Chest Diseases, Dow University of Health Sciences, Karachi, during July 2016 to December 2017. Sputum-scarce patients with suspicion of pulmonary tuberculosis were selected and underwent for bronchoscopy, detailed examination of bronchial tree was performed, and bronchial washing collected for testing of mycobacterium tuberculosis with Gene Xpert.

**Results:**

A total of 120 patients were included. In this study 55 (45.8%) patients were female and 65 (54.2%) were male with mean±SD of age was 39.9 ±14.7 years. Bronchial washing Gene Xpert for mycobacterium tuberculosis was detected in 83 (69.2%) sputum-scarce cases of suspected pulmonary tuberculosis patients.

**Conclusion:**

Bronchial washing Gene Xpert has an excellent diagnostic yield for detection of mycobacterium tuberculosis in sputum-scarce cases of suspected pulmonary tuberculosis.

## INTRODUCTION

Pulmonary Tuberculosis (PTB) is a major public health problem, affecting the population world widely. *Mycobacterium tuberculosis* (MTB); a type of airborne bacterium is a causative agent for PTB.^1^,^2^ Unfortunately, PTB affects one third population of the world including one third patients with active PTB.^3^ In year 2014, the World Health Organization (WHO) published a global tuberculosis (TB) report, which showed 10.4 million new cases of PTB in year 2014. Tuberculosis remains a common public health hazard in the underdeveloped country like Pakistan, which is included in the top six nations of the world that makes 60% of new PTB cases.^4^

PTB diagnosis is based on isolation of the organism from respiratory specimens, especially sputum samples. WHO suggests the bacteriological identification and confirmation of PTB by means of acid-fast bacilli (AFB) in respiratory specimens.^1^,^4^ Several tests are available to confirm the disease.

Smear microscopy is still gold standard in many developing countries, but up to 22.8% cases are smear negative and has higher mortality as compared with smear positive.^5^ Culture of MTB has long incubation time and limits its use in early diagnosis.^6^ Only about 20-40% of PTB patients are smear positive, while the rest of the patients had either smear negative or sputum-scarce disease.^7^-^9^ Thus, diagnosis in 60-80% of patients is not confirmed by routine tests. It is imperative to confirm diagnosis of PTB in these patients. Bronchoscopy with bronchial washing (BW) is needed for sputum-scarce patients to establish the diagnosis.^10^

The rational of this study was to determine the frequency of detection of Mycobacterium tuberculosis in bronchial washing Gene Xpert in sputum-scarce cases of suspected PTB. The utilization of this study is to identify the number of cases that are sputum-scarce yet having PTB. If the frequency of MTB on BW found to be high, not only complications related to MTB can be reduced but so is the associated morbidity and mortality. Early screening of sputum-scarce cases will help to prevent the associated complications and early management can be practiced. Local data is scarce regarding this study in the population of Sindh. This research was designed to find out the current magnitude of MTB in sputum-scarce cases. Our objective was to determine the frequency of mycobacterium tuberculosis detection, in bronchial washing in sputum-scarce cases of suspected pulmonary tuberculosis.

## METHODS

This cross-sectional research was designed and conducted at a tertiary care teaching hospital, during July 2016 to December 2017. Non-probability consecutive method was used for selection of patients. The ethical approval of study was done from Institutional Review Board of our University.

### Inclusion Criteria

Patients having age between 18 to 60 years, either male or female, with suspected PTB with sputum-scarce as per operational definitions, taking anti-TB medications less than 1 week, and naive were included in the study.

### Exclusion Criteria

Patients with disseminated or extra pulmonary tuberculosis, HIV positive and immunocompromised patients were excluded from the study.

A written informed consent was obtained from the patient undergoing bronchoscopy procedure. Patients fulfilling the inclusion criteria were selected. Taking detailed history regarding cough, fever and loss of weight, thorough physical examination and routine investigations, assessment for the fitness of patients for the bronchoscopy procedure was performed. Details regarding educational status were recorded. Bronchoscope was introduced through trans-nasal route after proper lubrication with xylocaine ointment. Detailed examination of bronchial tree was performed, and BW was collected and sent to the laboratory for Gene Xpert, and presence or absence of MTB was recorded. For data analysis Statistical Package for Social Science (SPSS) software version 22 was used. P-value of ≤0.05 was taken as significant.

Sample size was calculated by using the proportions as reported by Khalil KF et al.^11^, 93 patients with suspected PTB who were sputum-sarce were subjected to bronchoscopy. PTB was confirmed by bronchial wash Gene Xpert in 81, p = 87.09% of them, with 95% confidential interval and 6% of margin of error, the sample size stands n = 120.

### Operational Definition Suspected PTB

PTB diagnosis is suspected from a combination of:

### Symptoms

Cough with sputum, fever (more than 98.4 °F), sweating and shortness of breathing

### Signs

Respiratory rate more than 20 per minute, weight loss (>*5%* of body weight over 6 to 12 months)

### Chest Radiograph

Consolidation, Cavitation, Nodular opacity and military shadows.

Presence of ≥ 2 clinical features of > 2 weeks duration with any of these chest X-ray findings in smear negative cases, are known as Suspected Pulmonary Tuberculosis cases.

### Sputum-Scarce patients^11^

Patients that had sputum amount less than 1 ml were defined to have sputum-Scarce patients.

## RESULTS

In this study 120 patients were included. Mean±SD of age was 39.9 ±14.7 years, duration of symptoms was 5.4 ±2.9 weeks and duration of ATT was 3.5 ±3.1days. Out of 120 selected patients 55 (45.8%) patients were female and 65 (54.2%) were male. Selected patients were segregated into following age groups; 18-30 years 44 (36.7%) patients, 31-40 years 18(15%) patients, 41-50 years 21 (17.5%) patients and 51-60 years 37 (30.8%) patients. Socio-economic status of selected patients was; lower class 45 (37.5%) patients, middle class 69 (57.5%) patients and upper class 6 (5%) patients. Educational status was; illiterate 29 (24.2%), primary 10 (8.3%), middle 5 (4.2%), matric 22 (18.3%), intermediate 36 (30%) and graduate/post graduate 18 (15%).

Duration of symptoms in selected patients was; 2-8 weeks in 107 (89.2%) patients and 9-16 weeks in 13 (10.8%) patients. Duration of Anti Tuberculosis Therapy (ATT) in selected patients was; Naive 51 (42.5%) patients, 1-4 days 7 (5.8%) patients and 5-7 days 62 (51.7%) patients. Gene Xpert MTB was detected in 83 (69.2%) patients. Thus, PTB was confirmed in 69.2% of patients who were sputum-scarce. 4 (3.3%) patients were found to have rifampicin resistant.

Bronchial Wash Gene Xpert for MTB results were cross tabulated with different effect modifiers such as gender, age, socio-economic status, educational status, duration of symptoms and ATT. Differences in frequency according to these effect modifiers was assessed by χ^2^ test. No significant difference was observed in frequency according to the effect modifiers. Details are given in Table-I.

**Table-I:** Stratification of Bronchial Wash Gene Xpert for MTB^1^ with different variables.

Variables	Bronchial Wash Gene Xpert for MTB		Total	P-Value

	Detected	Not Detected		
*Gender*				
Male	45	20	65	0.987
Female	38	17	55	
*Age Groups (Years)*				
18-30	29	15	44	0.785
31-40	12	6	18	
41-50	14	7	21	
51-60	28	9	37	
*Socio-economic Status*				
Lower Class	31	14	45	0.564
Middle Class	49	20	69	
Upper Class	3	3	6	
*Educational Status*				
Illiterate	18	11	29	0.364
Primary	9	1	10	
Middle	5	0	5	
Matric	14	8	22	
Intermediate	24	12	36	
Graduate/Post-graduate	13	5	18	
*Duration of Symptoms*				
2-8 Weeks	73	34	107	0.521
9-16 Weeks	10	3	13	
*Duration of Anti-tuberculous therapy*				
Naïve	32	19	51	0.329
1-4 Days	6	1	7	
5-7 Days	45	17	62	

MTB = Mycobacterium Tuberculosis

## DISCUSSION

Tuberculosis is still a major global health problem, affecting millions every year in developing countries. TB is ranked as the second leading cause of death worldwide from an infectious cause, after HIV. TB mortality remains unacceptably high given that most deaths are preventable, if they have access to health care for a timely diagnosis and the proper treatment.^12^

In our study, we determined the frequency of detection of Mycobacterium tuberculosis in BW Gene Xpert in sputum-sarce cases of suspected pulmonary tuberculosis. Many sputum smear negative patients are reported to have progressive disease.^13^,^14^ Previous reports in non-immune compromised patients with sputum sputum-scarce TB indicated that bronchoscope specimens and post bronchoscope sputum are helpful in establishing the diagnosis.^9^,^15^ Superiority of Gene Xpert in immunocompromised patients to detect TB as compared to microscopy is debatable. Yield of BW is also reported to be superior to brush microscopy. Similar results were demonstrated by different researchers, such as Lyer VN et al, Mohan A *et al* and Shin JA *et al reporting detection of TB in 30-48% in smear negative patients*.^16^-^18^ Different studies validated the practical and clinical importance of bronchoscopy in PTB diagnosis.^10^,^15^,^16^,^18^,^19^ In all these studies bronchoscopies and BW showed increased detection of TB.

Bronchoscopy with BW under local anesthesia is a relatively safe procedure and well tolerated by most of the patients. Complications are rare in occurrence.^20^ As all patients are sputum-scarce, delay in diagnosis in this subset often leads to increased morbidity and mortality, BW Gene Xpert can rapidly detect the MTB and rule out rifampicin resistance on the same day, helping in the early diagnosis and management of these patients.^21^-^23^ In our study 37 patients were negative for MTB on BW Gene Xpert and such patients should be subjected to AFB culture to confirm PTB.

## CONCLUSION

Bronchial washing Gene Xpert has an excellent diagnostic yield for detection of MTB in sputum-scarce cases of suspected PTB. In our study we were able to make diagnosis of PTB in 69.2% of patients who were not able to produce sputum.

### Authors’ Contribution

**SHu** did data collection; initial manuscript write up.

**FFZ** conceived and designed study, manuscript editing and final approval.

**SHa** did data collection and data analysis and initial manuscript write-up.

**BFZ** did manuscript corrections, revision and statistical analysis.

## References

[1] Khan WM, Smith H, Qadeer E, Hassounah S (2016). Knowledge and perceptions of national and provincial tuberculosis control programme managers in Pakistan about the WHO Stop TB strategy:a qualitative study. JRSM Open.

[2] Stop TB (2008). Policy Paper:Contributing to Health System Strengthening:Guiding Principles for National Tuberculosis Programmes.

[3] (2012). WHO Policy on Collaborative TB/HIV Activities:Guidelines for National Programmes and Other Stakeholders.

[4] Zumla A, George A, Sharma V, Herbert RH, Baroness Masham of I, Oxley A (2015). The WHO 2014 global tuberculosis report--further to go. Lancet Glob Health.

[5] Pourostadi MMP, Rashedi JM, Mahdavi Poor BM, Samadi Kafil HP, Hariri-Akbari MM, Asgharzadeh MP (2018). Frequency of Smear-Negative Tuberculosis in Northwest Iran. Iran J Med Sci.

[6] Ji L, Lou YL, Wu ZX, Jiang JQ, Fan XL, Wang LF (2017). Usefulness of interferon-gamma release assay for the diagnosis of sputum smear-negative pulmonary and extra-pulmonary TB in Zhejiang Province, China. Infect Dis Poverty.

[7] Chavalertsakul K, Boonsarngsuk V, Saengsri S, Santanirand P (2017). TB-PCR and drug resistance pattern in BALF in smear-negative active pulmonary TB. Int J Tuberc Lung Dis.

[8] Singh BK, Sharma SK, Sharma R, Sreenivas V, Myneedu VP, Kohli M (2017). Diagnostic utility of a line probe assay for multidrug resistant-TB in smear-negative pulmonary tuberculosis. PLoS One.

[9] Hermans SM, Babirye JA, Mbabazi O, Kakooza F, Colebunders R, Castelnuovo B (2017). Treatment decisions and mortality in HIV-positive presumptive smear-negative TB in the Xpert MTB/RIF era:a cohort study. BMC Infect Dis.

[10] George PM, Mehta M, Dhariwal J, Singanayagam A, Raphael CE, Salmasi M (2011). Post-bronchoscopy sputum:improving the diagnostic yield in smear negative pulmonary TB. Respir Med.

[11] Khalil KF, Butt T (2015). Diagnostic yield of Bronchoalveolar Lavage gene Xpert in smear-negative and sputum-scarce pulmonary tuberculosis. J Coll Physicians Surg Pak.

[12] Meaza A, Kebede A, Yaregal Z, Dagne Z, Moga S, Yenew B (2017). Evaluation of genotype MTBDRplus VER 2.0 line probe assay for the detection of MDR-TB in smear positive and negative sputum samples. BMC Infect Dis.

[13] Chadha VK, Praseeja P, Hemanthkumar NK, Shivshankara BA, Sharada MA, Nagendra N (2014). Are registered sputum smear-negative tuberculosis patients in Karnataka, India, diagnosed by national algorithm?. Int J Tuberc Lung Dis.

[14] Chadha VK, Praseeja P, Hemanthkumar NK, Shivshankara BA, Sharada MA, Nagendra N (2014). Implementation efficiency of a diagnostic algorithm in sputum smear-negative presumptive tuberculosis patients. Int J Tuberc Lung Dis.

[15] Patil S, Narwade S, Mirza M (2017). Bronchial Wash Gene Xpert MTB/RIF in Lower Lung Field Tuberculosis:Sensitive, Superior, and Rapid in Comparison with Conventional Diagnostic Techniques. J Transl Int Med.

[16] Iyer VN, Joshi AY, Boyce TG, Brutinel MW, Scalcini MC, Wilson JW (2011). Bronchoscopy in suspected pulmonary TB with negative induced-sputum smear and MTD((R)) Gen-probe testing. Respir Med.

[17] Mohan A, Sharma SK (2008). Fibreoptic bronchoscopy in the diagnosis of sputum smear-negative pulmonary tuberculosis:current status. Indian J Chest Dis Allied Sci.

[18] Shin JA, Chang YS, Kim TH, Kim HJ, Ahn CM, Byun MK (2012). Fiberoptic bronchoscopy for the rapid diagnosis of smear-negative pulmonary tuberculosis. BMC Infect Dis.

[19] Li X, Xu H, Jiang S, Jing K, Wang L, Liu X (2011). TB-SA antibody test for diagnosis and monitoring treatment outcome of sputum smear negative pulmonary tuberculosis patients. Southeast Asian J Trop Med Public Health.

[20] Hsu LH, Liu CC, Ko JS, Chen CC, Feng AC (2016). Safety of interventional bronchoscopy through complication review at a cancer center. Clin Respir J.

[21] Pan X, Yang S, Deighton MA, Qu Y, Hong L, Su F (2018). A Comprehensive Evaluation of Xpert MTB/RIF Assay With Bronchoalveolar Lavage Fluid as a Single Test or Combined With Conventional Assays for Diagnosis of Pulmonary Tuberculosis in China:A Two-Center Prospective Study. Front Microbiol.

[22] Lu Y, Zhu Y, Shen N, Tian L, Sun Z (2018). Evaluating the diagnostic accuracy of the Xpert MTB/RIF assay on bronchoalveolar lavage fluid:A retrospective study. Int J Infect Dis.

[23] Hong J, Lee SH, Ryu BH, Kim MJ, Jo KW, Chong YP (2018). Diagnostic usefulness of bronchoalveolar lavage fluid xpert MTB/RIF in pauci-bacillary pulmonary tuberculosis. Infect Dis (Lond).

